# Schambezogene Störungen bei Patienten mit atopischer Dermatitis und Psoriasis – eine explorative, Querschnitts‐Interviewstudie zu Prävalenz und Korrelaten körperdysmorpher Störungen und sozialer Angststörungen

**DOI:** 10.1111/ddg.15892_g

**Published:** 2026-02-05

**Authors:** Clara Wülfing, Carsten Spitzer, Laura Lübke, Steffen Emmert, Alexander Thiem

**Affiliations:** ^1^ Klinik und Poliklinik für Psychosomatische Medizin und Psychotherapie Universitätsmedizin Rostock; ^2^ Klinik und Poliklinik für Dermatologie und Venerologie Universitätsmedizin Rostock

**Keywords:** Atopische Dermatitis, körperdysmorphe Störung, Psoriasis, Querschnittsstudie, schambezogene Störungen, soziale Phobie, Atopic dermatitis, body dysmorphic disorder, cross‐sectional study, psoriasis, shame‐related disorders, social anxiety disorder

## Abstract

**Hintergrund und Ziele:**

Atopische Dermatitis (AD) und Psoriasis (Pso) gehen oft mit psychischer Belastung einher. In dieser Studie untersuchten wir die Prävalenz und die Korrelate schambezogener Störungen (SRD), das sind die körperdysmorphe Störung (BDD) und die soziale Angststörung (SAD), bei Patienten mit AD und Pso.

**Patienten und Methodik:**

In einer monozentrischen Querschnittsstudie wurden zwischen 07/2023 und 03/2024 erwachsene Patienten rekrutiert. Eine klinische Psychologin führte diagnostische Interviews zur Erfassung von BDD und SAD durch. Zusätzlich wurden der DLQI und der EASI beziehungsweise der PASI erhoben.

**Ergebnisse:**

Insgesamt wurden 151 Patienten eingeschlossen, davon 55 (36,4%) mit AD und 96 (63,6%) mit Pso. Die Punkt‐ und Lebenszeitprävalenz von SRD betrugen in der Gesamtstichprobe 17,2% beziehungsweise 31,8%. Für BDD lag die Punktprävalenz bei 10,6%, die Lebenszeitprävalenz bei 26,5%. Für SAD betrugen diese Werte 12,6% beziehungsweise 17,2%. Es zeigten sich keine signifikanten Unterschiede zwischen Patienten mit AD und Pso. SRD waren signifikant mit jüngerem Alter und weiblichem Geschlecht assoziiert. Zudem war der DLQI bei Betroffenen mit SRD deutlich eingeschränkt.

**Schlussfolgerungen:**

Die Ergebnisse dieser Studie zeigen, dass SRD bei Patienten mit AD und Pso häufig vorkommen und ihre routinemäßige Erfassung in der Klinik weiter untersucht werden sollte.

## EINLEITUNG

Atopische Dermatitis (AD) und Psoriasis (Pso) gehören zu den häufigsten chronisch‐entzündlichen Hauterkrankungen und betreffen weltweit zwischen 2% (Pso) und 4,5% (AD) der erwachsenen Allgemeinbevölkerung.^1^, ^2^ Aufgrund ihres chronischen, häufig schubförmigen Verlaufs sind beide Erkrankungen mit einer erheblichen Krankheitslast verbunden, darunter intensiver Juckreiz, Schlafstörungen, eingeschränkte psychosoziale Funktionsfähigkeit, reduzierte Lebensqualität (QoL) und schlechte psychische Gesundheit.^3^, ^4^, ^5^, ^6^ Darüber hinaus erleben viele Menschen mit AD oder Pso Stigmatisierung und Diskriminierung.^7^, ^8^ Diese Faktoren können sich gegenseitig negativ verstärken und zu einer kumulativen Beeinträchtigung im Lebensverlauf führen,^9^ was wiederum mit psychischer Belastung sowie negativen Affekten, wie Depression, Angst und Scham, einhergehen kann.

Metaanalysen zeigen eine signifikante Assoziation zwischen AD bei Erwachsenen und Depression sowie Angststörungen.^10^, ^11^, ^12^ Beispielsweise hatten Patienten mit AD in einer multizentrischen Studie mit 162 Teilnehmenden aus 13 europäischen Ländern eine mehr als dreifach höhere Wahrscheinlichkeit klinisch depressiv zu sein als eine Kontrollgruppe.^13^ Langzeitstudien weisen darauf hin, dass erwachsene Patienten mit AD ein erhöhtes Risiko für die Entwicklung einer *major depression* oder anderen depressiven Störungen haben.^14^ Zudem zeigen Metaanalysen einen Zusammenhang zwischen der Schwere der AD und depressiven Symptomen.^10^ Genauso treten klinisch‐relevante Angst und Angststörungen bei Erwachsenen mit AD gehäuft auf.^13^, ^15^

Auch für Psoriasis zeigen systematische Übersichtsarbeiten und Metaanalysen eine klare Assoziation mit Depression und Angst.^15^, ^16^, ^17^, ^18^ Allerdings variiert die Prävalenz depressiver Störungen bei Erwachsenen mit Pso erheblich, insbesondere aufgrund methodischer Unterschiede in der Depressionsdiagnostik.^18^ Studien mit klinischen Interviewberichten zeigten in der Regel niedrigere Prävalenzraten als Studien mit Selbstberichtsfragebögen.^18^ Ein ähnliches Bild zeigt sich für Angststörungen bei Pso: Prävalenzschätzungen fallen höher aus, wenn Selbstbeurteilungsinstrumente verwendet werden, als wenn diagnostische Interviews durchgeführt werden.^15^, ^17^ Zudem wurde berichtet, dass die Schwere der Pso mit erhöhten Werten für Depression und Angst korreliert.^15^, ^16^, ^17^

Während Depression und Angststörungen gut untersucht sind, findet das Thema Scham in der Dermatologie erst allmählich wissenschaftliche Beachtung.^19^, ^20^ Besonders relevant in diesem Zusammenhang sind die körperdysmorphe Störung (*body dysmorphic disorder*; BDD) und die soziale Angststörung (social anxiety disorder; SAD), die als schambezogene Störungen (*shame‐related disorders*, SRD) eingeordnet werden.^21^, ^22^

Die körperdysmorphe Störung (BDD) ist gekennzeichnet durch eine anhaltende und belastende Beschäftigung mit einem vermeintlichen oder geringfügigen Makel des äußeren Erscheinungsbilds, wobei bestehende Abweichungen stark übertrieben wahrgenommen werden. Diese übermäßige Fixierung führt zu erheblichem Leidensdruck sowie Einschränkungen in der Lebensqualität und psychosozialen Funktionsfähigkeit.^23^ Obwohl BDD in der Dermatologie zunehmend erforscht wird,^24^, ^25^ fehlen systematische Erkenntnisse zur Prävalenz und den Korrelaten von BDD bei Patienten mit AD oder Pso.

Eine aktuelle multizentrische Querschnittsstudie mit 5487 konsekutiven dermatologischen Patienten in 17 europäischen Ländern zeigte, dass erwachsene Patienten mit AD oder Pso ein mehr als siebenfach erhöhtes Risiko für selbstberichtete BDD‐Symptome im Vergleich zu gesunden Kontrollpersonen hatten.^26^ Die geschätzte Prävalenz von BDD betrug 15,9% für AD‐Patienten und 13,9% für Pso‐Patienten. In ambulanten dermatologischen Patienten im Allgemeinen war BDD insgesamt signifikant mit jüngerem Alter, weiblichem Geschlecht und einer reduzierten Lebensqualität assoziiert.^24^, ^26^

Das Hauptmerkmal der SAD ist eine übermäßige Angst vor sozialen oder leistungsbezogenen Situationen, in denen Beschämung oder Ablehnung befürchtet wird, was zu Vermeidungsverhalten und erheblichen Einschränkungen in verschiedenen Lebensbereichen führt.^27^ Die Befunde zu SAD bei erwachsenen Patienten mit Pso sind inkonsistent, wie eine aktuelle Metaanalyse zeigt.^28^ Während einige Studien eine hohe Prävalenz von SAD berichten, bestehen erhebliche methodische Unterschiede, die zu einer breiten Spannweite an Prävalenzschätzungen führen: Sie reichen von 3% in Studien mit diagnostischen Interviews bis zu 42% in Studien mit Selbstberichtsfragebögen.^28^ Trotz umfangreicher Forschung zu Angststörungen bei AD,^13^, ^15^ ist die Prävalenz von SAD bei AD‐Patienten bisher nicht bekannt.

Zusammenfassend lässt sich feststellen, dass die Forschung zu schambezogenen Störungen (BDD und SAD) bei erwachsenen Patienten mit AD und Pso bisher begrenzt, uneinheitlich und möglicherweise durch methodische Verzerrungen in Selbstberichtsmessungen beeinträchtigt ist. Vor dem Hintergrund dieser Befunde verfolgt unsere explorative Studie zwei zentrale Forschungsfragen: *(1)* Wie hoch sind die Punkt‐ und Lebenszeitprävalenz von BDD und SAD bei erwachsenen Patienten mit AD oder Pso, wenn halbstrukturierte Interviews zur Diagnosestellung verwendet werden? *(2)* Wie hängen Lebenszeitdiagnosen von BDD und SAD mit Alter, Geschlecht, Krankheitsdauer und ‐schwere, gesundheitsbezogener Lebensqualität und aktueller Behandlung zusammen?

## STICHPROBE UND METHODIK

### Studiendesign und Rekrutierung

Die Datenerhebung erfolgte während dermatologischer Vorstellungen in der Ambulanz der Klinik für Dermatologie und Venerologie am Universitätsklinikum Rostock, Deutschland. Zur Studienteilnahme waren ein Mindestalter von 18 Jahren, eine dermatologisch bestätigte Diagnose einer AD oder Pso, das Fehlen einer kognitiven Beeinträchtigung sowie ausreichende Deutschkenntnisse zur Beantwortung der Interviewfragen erforderlich. Patienten mit ausschließlich pustulöser, palmoplantarer, guttater oder Nagelpsoriasis wurden von der Studie ausgeschlossen.

Allen geeigneten Patienten wurde die Teilnahme angeboten. Nach ausführlicher Aufklärung über das Studiendesign und schriftlicher Einwilligung wurden die Teilnehmer von einer geschulten klinischen Psychologin (C.W.) diagnostisch interviewt. Im Anschluss füllten sie den *Dermatology Life Quality Index* (DLQI; siehe unten) aus. Demografische Daten wurden mittels Selbstauskunft erhoben. Der behandelnde Dermatologe dokumentierte die Schwere und Lokalisation der Hauterkrankung anhand des *Psoriasis Area and Severity Index* (PASI)^29^, ^30^ für Pso oder des *Eczema Area and Severity Index* (EASI)^31^ für AD. Diese Querschnitts‐ Beobachtungsstudie wurde von der lokalen Ethikkommission genehmigt (Genehmigungsnummer A 2023‐0051) und entsprach den Kriterien der Deklaration von Helsinki. Der Studienzeitraum erstreckte sich von Juli 2023 bis März 2024. Von 217 geeigneten Patienten, darunter 148 mit Pso und 69 mit AD, willigten 163 Personen (75,1%) in die Studienteilnahme ein. Aufgrund fehlender Daten oder eines nachträglichen Widerrufs der Einwilligung mussten zwölf Patienten ausgeschlossen werden, sodass die finale Stichprobe 151 Patienten umfasste, darunter 96 mit Pso und 55 mit AD.

### Instrumente

Die Erfassung schambezogener Störungen erfolgte in einem zweistufigen Verfahren. Zu Beginn des Interviews wurden körperdysmorphe Sorgen und soziale Ängste anhand der Screening‐Fragen des *Structured Clinical Interview for DSM‐5 Disorders – Clinician Version* (SCID‐5‐CV)^32^ erhoben. Die Frage zur körperdysmorphen Störung (BDD) lautete: „Hatten Sie jemals große Sorgen, dass etwas mit Ihrem äußeren Erscheinungsbild nicht stimmt oder dass ein bestimmter Körperbereich unvorteilhaft aussieht?“, während soziale Ängste mit folgender Frage erfasst wurden: „Waren Sie jemals in sozialen Situationen – beispielsweise in Gesprächen oder beim Treffen mit unbekannten Personen – besonders nervös oder ängstlich?“ Wurde eine dieser Fragen bejaht, folgte eine detaillierte Diagnostik zur Erfassung der Punkt‐ und Lebenszeitprävalenz von BDD und SAD. Eine SRD wurde definiert als das Vorliegen von BDD oder SAD, sowohl lebenszeitlich als auch aktuell.

#### Yale‐Brown Obsessive‐Compulsive Scale – Modifizierte Version für die körperdysmorphe Störung (BDD‐YBOCS)

Symptome der BDD wurden mit der deutschen Version der *Yale‐Brown Obsessive‐Compulsive Scale – modifiziert für die körperdysmorphe Störung* (BDD‐YBOCS)^33^, ^34^, ^35^ – erfasst, einem 12‐Item umfassenden klinischen Interview. Die BDD‐YBOCS erfasst die gedankliche Fixierung auf vermeintliche Makel, damit verbundene zwanghafte Verhaltensweisen, Vermeidungsverhalten und die Einsichtsfähigkeit. Jedes Item wird von 0 (keine Symptome) bis 4 (extreme Symptome) bewertet, wodurch sich ein Gesamtscore zwischen 0 und 48 Punkten ergibt. Ein Cut‐off‐Wert von 20 oder höher weist auf eine klinisch relevante BDD hin.^34^, ^36^ Die psychometrischen Eigenschaften der BDD‐YBOCS wurden als gut bis exzellent beschrieben.^34^, ^37^ In der vorliegenden Studie wurde neben der üblichen Zeitspanne der letzten Woche auch die gesamte Lebensspanne berücksichtigt.

#### Liebowitz Social Anxiety Scale (LSAS)

Die LSAS (Liebowitz, 1987; deutsche Version von von Consbruch et al.^38^) ist ein klinisch geführtes, semistrukturiertes Interview zur Beurteilung von SAD‐Symptomen. Sie umfasst zwei Subskalen zu sozialen Interaktionen (11 Items) und leistungsbezogenen Situationen (13 Items), die das individuelle Ausmaß an Angst und Vermeidung in sozialen Kontexten messen. Die Antworten werden auf einer vierstufigen Likert‐Skala gegeben. Jedes Item wird getrennt für Angst‐ und Vermeidungsverhalten erfasst, sodass der Gesamtscore zwischen 0 und 144 Punkten liegen kann. Ein Wert von über 30 unterscheidet zuverlässig zwischen Personen mit und ohne soziale Angststörung.^39^ Frühere Studien haben robuste psychometrische Eigenschaften der LSAS nachgewiesen.^40^ Auch für diese Skala wurden sowohl die Punktprävalenz (Symptome in der letzten Woche) als auch die Lebenszeitprävalenz erfasst.

#### Dermatology Life Quality Index (DLQI)

Der DLQI ist ein etabliertes, generisches Selbstbeurteilungsinstrument zur Erfassung der gesundheitsbezogenen Lebensqualität in der vergangenen Woche bei Patienten mit Hauterkrankungen.^41^ Er besteht aus zehn Items, die auf einer vierstufigen Likert‐Skala bewertet werden. Der Gesamtscore reicht von 0 bis 30 Punkten, wobei höhere Werte eine stärkere Beeinträchtigung der Lebensqualität anzeigen. Die psychometrischen Eigenschaften des DLQI wurden in zahlreichen Studien bestätigt.^42^

### Statistische Analysen

Neben deskriptiven Analysen wurden Gruppenvergleiche zwischen AD‐ und Pso‐Patienten hinsichtlich schambezogener Störungen durchgeführt. Für kategoriale Variablen wurde der χ^2^‐Test verwendet, während für metrische Variablen, wie beispielsweise die DLQI‐Daten, der nichtparametrische Mann‐Whitney‐U‐Test zur Anwendung kam. Letzterer wurde gewählt, um Verzerrungen durch kleine oder ungleich verteilte Stichproben zu vermeiden. Das Signifikanzniveau wurde auf α < 0,05 festgelegt. Alle statistischen Analysen wurden mit dem *Statistical Package for the Social Sciences* (SPSS, Version 27) durchgeführt.

## ERGEBNISSE

### Studienpopulation

Die Studienpopulation bestand aus insgesamt 151 Personen, darunter 65 Frauen (43,0 %) und 86 Männer (57,0 %), mit einem durchschnittlichen Alter von 45,2 Jahren (Standardabweichung 16,2; Bereich 18–85 Jahre). Von den Teilnehmern litten 55 (36,4%) an einer AD und 96 (63,6%) an Pso. Eine detaillierte Darstellung der soziodemografischen und klinischen Charakteristika, einschließlich der aktuellen Behandlung, ist in Tabelle 1 zu finden.

**TABELLE 1:** Soziodemografische und klinische Charakteristika der Studienpopulation.

	Gesamt (n = 151)	AD‐Patienten (n = 55)	Pso‐Patienten (n = 96)
***Alter*** (in Jahren; MW ± SD; Bereich)	45,2 ± 16,2 (18–85)	40,2 ± 16,6 (18–80)	48,0 ± 15,3 (19–85)
***Geschlecht***			
Frauen	65 (43,0%)	26 (47,3%)	39 (40,6%)
Männer	86 (57,0%)	29 (52,7%)	57 (59,4%)
***Familienstand***			
alleinstehend, kein Partner	56 (37,1%)	26 (47,3%)	30 (31,3%)
verheiratet/in fester Partnerschaft	78 (51,7%)	20 (36,4%)	58 (60,4%)
geschieden/getrennt lebend	17 (11,3%)	9 (16,4%)	8 (8,3%)
***Schulabschluss***			
Abitur	88 (58,3%)	28 (50,9%)	60 (62,5%)
Mittlere Reife	53 (35,1%)	23 (41,8%)	30 (31,3%)
andere	10 (6,6%)	4 (7,3%)	6 (6,3%)
***Krankheitsdauer*** (in Jahren; MW ± SD; Bereich)	23,0 ± 16,7 (0–64)	27,3 ± 16,4 (0–64)	20,6 ± 16,4 (0–58)
***Sichtbare Läsion*** (ja)	94 (63,1%)	40 (75,5%)	54 (56,3%)
***Schweregrad der Erkrankung***			
EASI (MW ± SD)		5,74 ± 7,59	–
PASI (MW ± SD)		–	3,33 ± 6,02
***DLQI*** (MW ± SD)	5,05 ± 6,21	6,51 ± 6,88	4,21 ± 5,65
***Aktuelle Behandlung***			
keine Behandlung	5 (3,3%)	1 (1,8%)	4 (4,2%)
nur Topika	24 (15,9%)	10 (18,2%)	14 (14,6%)
nur Biologika	55 (36,4%)	11 (20,0%)	44 (45,8%)
andere	14 (9,3%)	2 (3,6%)	12 (12,5%)
Topika und Andere	15 (9,9%)	6 (10,9%)	9 (9,4%)
Biologika und Topika	34 (22,5%)	23 (41,8%)	11 (11,5%)
Biologika und Andere	2 (1,3%)	1 (1,8%)	1 (1,0%)
Biologika, Topika und andere	2 (1,3%)	1 (1,8%)	1 (1,0%)

*Abk*.: AD, atopische Dermatitis; DLQI, dermatologischer Lebensqualitätsindex; EASI, *eczema area severity index*; MW, Mittelwert; PASI, *psoriasis area severity index*; Pso, Psoriasis; SD, Standardabweichung; SRD, schambezogene Störungen

Zu den topischen Therapien gehören topisch angewandte entzündungshemmende Medikamente wie Steroide als Monotherapie oder in Kombination mit Vitamin‐D‐Analoga. Zu den weiteren Behandlungsoptionen zählen orale Medikamente wie JAK‐Inhibitoren sowie UV‐Phototherapie.

### Prävalenz von BDD und SAD

**ABBILDUNG 1:**
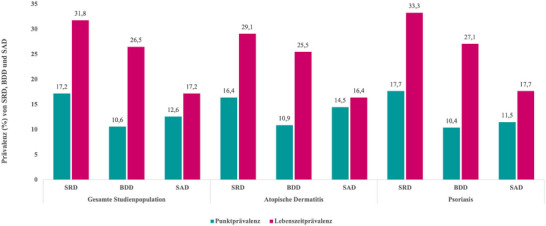
Vergleich der Punkt‐ und Lebenszeitprävalenz von schambezogenen Störungen (SRD), körperdysmorpher Störung (BDD) und sozialer Angststörung (SAD) bei Patienten mit chronisch‐entzündlichen Hauterkrankungen, atopischer Dermatitis (AD) oder Psoriasis (Pso). Es bestanden keine signifikanten Unterschiede in der Punkt‐ oder Lebenszeitprävalenz zwischen Patienten mit AD und Pso.

Unter allen Teilnehmern mit einer chronisch‐entzündlichen Hauterkrankung lag die Punktprävalenz schambezogener Störungen (SRD) bei 17,2% und die Lebenszeitprävalenz bei 31,8%. Die Prävalenzraten für die körperdysmorphe Störung (BDD) und die soziale Angststörung (SAD) bei Patienten mit Pso und AD sind in Abbildung 1 dargestellt. Es zeigten sich keine signifikanten Unterschiede in der Punkt‐ oder Lebenszeitprävalenz von BDD oder SAD zwischen den beiden Gruppen.

### Soziodemografische‐ und klinische Korrelate schambezogener Störungen

In der gesamten Studienpopulation war eine Lebenszeit‐Diagnose einer schambezogenen Störung (SRD) mit jüngerem Alter und weiblichem Geschlecht assoziiert. Dieses Muster zeigte sich sowohl für Patienten mit AD als auch für Patienten mit Pso (Tabelle 2). Weder die Krankheitsdauer noch die Sichtbarkeit der Hautläsionen waren mit dem Vorliegen einer SRD assoziiert. Ebenso bestand bei Patienten mit AD oder Pso kein Zusammenhang zwischen der Schwere der Hauterkrankung, gemessen anhand des EASI beziehungsweise PASI, und dem Auftreten einer SRD. Hinsichtlich der Lebensqualität zeigte sich, dass Patienten, die aktuell an einer schambezogenen Störung litten, signifikant niedrigere DLQI‐Werte aufwiesen. Dieser Effekt war sowohl in der Gesamtstichprobe als auch innerhalb der beiden Gruppen von Patienten mit AD oder Pso nachweisbar. Da Item 2 des DLQI explizit auf schambezogene Empfindungen in Bezug auf das Hautbild eingeht („Wie beschämt oder verlegen waren Sie in der vergangenen Woche aufgrund Ihrer Haut?“), untersuchten wir zusätzlich, ob der Zusammenhang zwischen SRD und DLQI auch bei Ausschluss dieses Items bestehen blieb. Es ergaben sich nahezu identische Korrelationen der DLQI‐Werte sowohl mit (ρ = 0,320, p < 0,001) als auch ohne Item 2 (ρ = 0,323, p < 0,001). Dieses Muster zeigte sich konsistent auch für die Korrelationen mit BDD und SAD (Daten nicht dargestellt).

**TABELLE 2:** Vergleich von Alter, Geschlecht, Schweregrad und Dauer der Erkrankung sowie Lebensqualität bei Patienten mit chronisch‐entzündlichen Hauterkrankungen mit und ohne schambezogene Störungen (SRD; Lebenszeit).

	SRD +	SRD –	Statistik	
	*Gesamtstichprobe*		*U/ χ^2^*	*p*
Alter (in Jahren, MW ± SD)	38,0 ± 15,9	48,5 ± 15,3	1532,0	*< 0,001*
Frauen	30 (62,5%)	35 (34,0%)	10,862	*< 0,001*
sichtbare Läsion	31 (64,6%)	63 (62,4%)	0,068	0,794
Krankheitsdauer (in Jahren; MW ± SD)	20,0 ± 15,2	24,4 ± 17,2	2026,0	0,152
DLQI	6,64 ± 6,93	4,33 ± 5,75	1804,0	*0,012*
	***Atopische Dermatitis***			
Alter (in Jahren, MW ± SD)	30,9 ± 11,4	44,0 ± 17,0	160,0	*0,005*
Frauen	11 (68,8%)	15 (38,5%)	4,176	*0,041*
EASI (MW ± SD)	5,64 ± 9,66	5,78 ± 6,71	273,0	0,469
sichtbare Läsion	11 (68,8%)	29 (78,4%)	0,559	0,455
Krankheitsdauer (in Jahren; MW ± SD	25,7 ± 14,6	28,0 ± 14,6	261,5	0,504
DLQI	9,25 ± 9,27	5,38 ± 5,38	253,0	0,272
	***Psoriasis***			
Alter (in Jahren, MW ± SD)	41,6 ± 16,8	51,3 ± 13,5	672,5	*0,006*
Frauen	19 (59,4%)	20 (31,3%)	6,996	*0,008*
PASI (MW ± SD)	3,97 ± 6,92	3,01 ± 5,55	897,5	0,316
sichtbare Läsion	20 (62,5%)	34 (53,1%)	0,762	0,383
Krankheitsdauer (in Jahren; MW ± SD)	17,1 ± 14,9	22,4 ± 17,0	821,5	0,176
DLQI	5.,9 ± 5,01	3,69 ± 5,91	677,5	*0,011*

*Abk*.: DLQI, dermatologischer Lebensqualitätsindex; EASI, *eczema area severity index*; MW, Mittelwert; PASI, *psoriasis area severity index*; SD, Standardabweichung; SRD, schambezogene Störungen

Eine gesonderte Analyse der Lebenszeitdiagnosen von BDD und SAD ergab ein weitgehend identisches Bild (Tabellen 3, 4). In der Gesamtstichprobe sowie innerhalb der Gruppen mit AD und Pso waren sowohl BDD als auch SAD mit jüngerem Alter und weiblichem Geschlecht assoziiert, jedoch nicht mit der Schwere der Hauterkrankung, der Krankheitsdauer oder der Sichtbarkeit der Hautläsionen. Eine Ausnahme zeigte sich bei Patienten mit AD, bei denen kein signifikanter Zusammenhang zwischen dem Geschlecht und dem Vorliegen einer SAD bestand (Tabelle 4). Die Befunde zur Lebensqualität waren inkonsistent. Während die DLQI‐Werte bei Personen mit einer BDD oder SAD in beiden Gruppen tendenziell erhöht waren, zeigte sich lediglich bei AD‐Patienten mit SAD ein signifikanter Unterschied im Vergleich zu AD‐Patienten ohne SAD.

**TABELLE 3:** Vergleich von Alter, Geschlecht, Schweregrad und Dauer der Erkrankung sowie Lebensqualität bei Patienten mit chronisch‐entzündlichen Hauterkrankungen mit und ohne körperdysmorphe Störung (BDD; Lebenszeit).

	BDD +	BDD –	Statistik	
	*Gesamtstichprobe*		*U/ χ^2^*	*p*
Alter (in Jahren, MW ± SD)	37,4 ± 16,5	48,0 ± 15,2	1366,0	*< 0,001*
Frauen	27 (67,5%)	38 (34,2%)	13,272	*< 0,001*
sichtbare Läsion	24 (60,0%)	70 (64,2%)	0,224	0,636
Krankheitsdauer (in Jahren; MW ± SD)	22,4 ± 15,1	23,3 ± 17,3	2107,0	0,936
DLQI	6,13 ± 7,02	4,67 ± 5,89	1835,5	0,154
	***Atopische Dermatitis***			
Alter (in Jahren, MW ± SD)	30,9 ± 12,0	43,3 ± 16,9	148,0	*0,007*
Frauen	11 (78,6%)	15 (36,6%)	7,381	*0,007*
EASI (MW ± SD)	5,14 ± 9,86	5,94 ± 6,78	230,0	0,270
sichtbare Läsion	9 (64,3%)	31 (79,5%)	1,286	0,257
Krankheitsdauer (in Jahren; MW ± SD	27,7 ± 13,8	27,2 ± 17,3	269,5	0,944
DLQI	8,14 ± 9,28	5,95 ± 5,89	270,0	0,741
	***Psoriasis***			
Alter (in Jahren, MW ± SD)	40,9 ± 17,6	50,7 ± 13,5	605,0	*0,012*
Frauen	16 (61,5%)	23 (32,9%)	6,465	*0,011*
PASI (MW ± SD)	3,95 ± 7,31	3,10 ± 5,51	831,0	0,507
sichtbare Läsion	15 (57,7%)	39 (55,7%)	0,030	0,862
Krankheitsdauer (in Jahren; MW ± SD)	19,4 ± 15,1	21,1 ± 17,0	852,5	0,849
DLQI	5,00 ± 5,27	3,93 ± 5,80	691,0	0,113

*Abk*.: DLQI, dermatologischer Lebensqualitätsindex; EASI, *eczema area severity index*; MW, Mittelwert; PASI, *psoriasis area severity index*; SD, Standardabweichung

**TABELLE 4:** Vergleich von Alter, Geschlecht, Schweregrad und Dauer der Erkrankung sowie Lebensqualität bei Patienten mit chronisch‐entzündlichen Hauterkrankungen mit und ohne soziale Angststörung (SAD; Lebenszeit).

	SAD +	SAD –	Statistik	
	*Gesamtstichprobe*		*U/ χ^2^*	*p*
Alter (in Jahren, MW ± SD)	35,9 ± 14,0	47,1 ± 16,0	977,0	*< 0,001*
Frauen	17 (65,4%)	48 (38,4%)	6,393	*0,011*
sichtbare Läsion	17 (65,4%)	77 (62,6%)	0,071	0,789
Krankheitsdauer (in Jahren; MW ± SD)	17,2 ± 14,5	24,3 ± 16,9	1204,0	0,054
DLQI	7,15 ± 7,65	4,11 ± 5,81	1241,5	0,063
	***Atopische Dermatitis***			
Alter (in Jahren, MW ± SD)	30,1 ± 11,9	42,1 ± 16,8	112,0	*0,030*
Frauen	5 (55,6%)	21 (45,7%)	0,296	0,586
EASI (MW ± SD)	8,77 ± 12,19	5,14 ± 6,37	173,0	0,439
sichtbare Läsion	8 (88,9%)	32 (69,6%)	1,417	0,234
Krankheitsdauer (in Jahren; MW ± SD	23,7 ± 15,8	28,1 ± 16,5	157,5	0,343
DLQI	12,78 ± 10,10	5,28 ± 5,41	116,5	*0,039*
	***Psoriasis***			
Alter (in Jahren, MW ± SD)	38,9 ± 14,4	50,0 ± 14,8	401,0	*0,009*
Frauen	12 (70,6%)	27 (34,2%)	7,689	*0,006*
PASI (MW ± SD)	3,48 ± 6,63	3,29 ± 5,93	589,0	0,420
sichtbare Läsion	9 (52,9%)	45 (57,0%)	0,092	0,762
Krankheitsdauer (in Jahren; MW ± SD)	13,8 ± 12,9	22,1 ± 16,8	476,5	0,070
DLQI	4,18 ± 3,63	4,21 ± 5,66	551,0	0,270

*Abk*.: DLQI, dermatologischer Lebensqualitätsindex; EASI, eczema area severity index; MW, Mittelwert; PASI, psoriasis area severity index; SD, Standardabweichung

Angesichts der insgesamt geringen Hauterkrankungsschwere in der Stichprobe wurden die Analysen erneut durchgeführt, nachdem die Studienpopulation in zwei Gruppen unterteilt wurde: eine mit milder Krankheitsausprägung (EASI ≤ 15 beziehungsweise PASI ≤ 10) und eine mit moderater bis schwerer Krankheitsausprägung (EASI > 15 beziehungsweise PASI > 10). Die detaillierten Ergebnisse sind in den Tabellen S1 bis S3 im Online‐Supplement dargestellt. Für die Gruppe mit milder Krankheitsausprägung ergaben sich im Wesentlichen dieselben Muster wie zuvor: Schambezogene Störungen waren mit jüngerem Alter und weiblichem Geschlecht assoziiert, jedoch nicht mit Krankheitsdauer, Krankheitsausprägung oder der Sichtbarkeit der Hautläsionen. Die Assoziation mit der Lebensqualität blieb inkonsistent. Die Stichprobe der Patienten mit moderater bis schwerer Krankheitsausprägung war mit sechs Patienten in der AD‐Gruppe und neun Patienten in der Pso‐Gruppe zu klein für eine statistische Analyse. Deskriptive Auswertungen zeigten jedoch ein vergleichbares Muster.

Da die aktuelle Behandlung innerhalb der Studienpopulation stark variierte, wurden die Analysen zu einem möglichen Zusammenhang zwischen der SRD‐Lebenszeitprävalenz und der Art der Behandlung auf zwei zentrale Vergleiche beschränkt: Erstens wurde untersucht, ob Unterschiede zwischen Patienten mit oder ohne Biologika bestehen. Zweitens wurde die Gruppe der Patienten, die mit Biologika behandelt wurden, mit jener verglichen, die ausschließlich topische Therapien erhielten. Diese Vergleiche wurden gewählt, da sie die häufigsten Behandlungsarten innerhalb der Stichprobe repräsentierten. Die detaillierten Ergebnisse sind in Tabelle S4 im Online‐Supplement dargestellt. Während Patienten, die mit Biologika behandelt wurden, numerisch höhere Lebenszeitprävalenzraten für SRD, BDD und SAD aufwiesen als Patienten ohne Biologika oder solche, die ausschließlich topische Therapien erhielten, waren diese Unterschiede statistisch nicht signifikant.

## DISKUSSION

Aufbauend auf früheren Studien, die die Relevanz von Scham bei dermatologischen Patienten hervorheben, untersuchte diese explorative Querschnittsstudie die Prävalenz der schambezogenen Störungen BDD und SAD bei Patienten mit AD und Pso. Nach unserem Kenntnisstand ist dies die erste Studie, die zur Erfassung von BDD ein halbstrukturiertes Interview einsetzte, das als Goldstandard gilt. Im Gegensatz dazu beruhen die meisten bisherigen Untersuchungen zu BDD bei dermatologischen Patienten ausschließlich auf Selbstberichtsverfahren.^24^, ^25^ Nur wenige Studien verwendeten klinisch geführte Interviews^43^ zur Diagnose von BDD, schlossen jedoch dermatologische Patientengruppen ganz allgemein ein und berichteten keine spezifischen Prävalenzraten für Patienten mit AD oder Pso. Darüber hinaus ist dies die erste Studie, die die Prävalenz von SAD bei Patienten mit AD systematisch erfasste, da hierzu bislang keine Untersuchungen vorliegen.

Die Lebenszeitprävalenz von BDD unter Patienten mit chronisch‐entzündlichen Hauterkrankungen lag bei 26,5%, ohne signifikante Unterschiede zwischen AD‐ und Pso‐Patienten. Diese Rate ist fast doppelt so hoch wie die in einer aktuellen Studie berichteten Schätzungen von 13,9% für Pso und 15,9% für AD,^26^ welche leicht über der Punktprävalenz unserer Studie liegen. Diese deutlichen Unterschiede könnten auf methodische Ungleichheiten in der BDD‐Diagnostik zurückzuführen sein: Während Schut et al.^26^ ein selbstberichtetes Screening‐Tool verwendeten, in dem die Teilnehmer angaben, ob sie jemals unter typischen BDD‐Symptomen gelitten hatten, wurde in unserer Studie ein halbstrukturiertes, klinisch geführtes Interview eingesetzt. Eine weitere Untersuchung mit demselben Interviewverfahren berichtete eine BDD‐Prävalenz von 36% unter dermatologischen Patienten insgesamt,^43^ was mehr als dreimal so hoch ist wie die 10,5% klinisch relevanter BDD‐Symptome bei 5487 Patienten mit häufigen dermatologischen Erkrankungen in der Studie von Schut et al.^26^ Neben der Art der Erhebungsmethode (Selbstbericht vs. klinische Befragung) könnten deshalb auch das verwendete Screening‐Instrument sowie verschiedene Antwortverzerrungen die tatsächlichen Prävalenzraten von BDD beeinflussen.^44^

Bezüglich der SAD ergaben sich eine Punktprävalenz von 12,6% und eine Lebenszeitprävalenz von 17,2% in der Gesamtstichprobe, wiederum ohne signifikante Unterschiede zwischen AD‐ und Pso‐Patienten. Da es sich hierbei um die erste Untersuchung zur SAD‐Prävalenz bei erwachsenen Patienten mit AD auf Basis eines klinischen Interviews handelt, lassen sich unsere Ergebnisse nicht direkt mit früheren Studien vergleichen. Bei Patienten mit einer Lebenszeitdiagnose einer AD wurde jedoch eine Punktprävalenz von 8,5% für eine unspezifizierte Angststörung berichtet.^45^ Zudem wurde ein Zusammenhang zwischen AD und der generalisierten Angststörung festgestellt, allerdings ohne Angaben zur konkreten Prävalenz.^46^

Im Gegensatz zur AD zeigt die Metaanalyse eine hohe SAD‐Prävalenz bei Pso‐Patienten, die über alle Studien hinweg auf durchschnittlich 15% geschätzt wurde.^28^ Dieser Wert liegt in ähnlicher Größenordnung wie die in unserer Stichprobe ermittelte Prävalenz bei Pso‐Patienten. Betrachtet man jedoch ausschließlich Studien, die SAD mittels Interviews diagnostizierten, liegt die gepoolte Prävalenz bei lediglich 3%,^28^ was nicht nur erheblich niedriger als unsere Ergebnisse ausfällt, sondern auch unter der SAD‐Prävalenz in der Allgemeinbevölkerung liegt.^27^ Diese Inkonsistenzen sind wahrscheinlich auf methodische Artefakte zurückzuführen. Insbesondere wurden in den genannten Studien überwiegend klinische Interviews oder das *Mini International Neuropsychiatric Interview* (MINI) eingesetzt, welches für die Erfassung von SAD als wenig sensitiv gilt.^47^, ^48^

Im Einklang mit früheren Untersuchungen^16^, ^24^, ^26^ zeigen unsere Ergebnisse, dass schambezogene Störungen bei Patienten mit chronisch‐entzündlichen Hauterkrankungen mit jüngerem Alter und weiblichem Geschlecht assoziiert waren. Wie erwartet und konsistent mit früheren Studien^24^, ^26^ wirkten sich sowohl BDD als auch SAD negativ auf die gesundheitsbezogene Lebensqualität aus. Weder die Schwere noch die Dauer der zugrunde liegenden Hauterkrankung war jedoch mit der Lebenszeitprävalenz schambezogener Störungen assoziiert. Dieses Ergebnis war insofern überraschend, als dass ein solcher Zusammenhang naheliegend erscheint, insbesondere da sowohl für AD^10^ als auch für Pso^15^, ^16^, ^17^ ein Zusammenhang zwischen Krankheitsschwere und Depressionen beziehungsweise Angstsymptomen berichtet wurde. Möglicherweise war die Stichprobengröße nicht ausreichend, um kleine bis mittlere Effekte zu detektieren, insbesondere in der AD‐Subgruppe. Eine weitere Erklärung und zugleich eine wesentliche Limitation unserer Studie könnte die insgesamt geringe Krankheitsaktivität^49^, ^50^ der untersuchten Patienten sein, was möglicherweise auf den Erfolg vorheriger und aktueller Therapien oder auf einen Selektionsbias zurückzuführen ist. Die Unterteilung der Stichprobe in Patienten mit milder vs. moderater bis schwerer Krankheitsausprägung führte jedoch zu keinen neuen Erkenntnissen.

Bemerkenswerterweise konnte keine Assoziation zwischen der Sichtbarkeit von Hautläsionen und schambezogenen Störungen festgestellt werden. Eine mögliche Interpretation wäre, dass die Anfälligkeit für BDD oder SAD unabhängig von potenziell schaminduzierenden Faktoren ist, was durch frühere Studien gestützt wird.^26^, ^43^ Zukünftige Forschung ist erforderlich, um diese Frage weiter zu untersuchen.

Obwohl unsere Studie einige Stärken aufweist, insbesondere die detaillierte diagnostische Erfassung durch ein halbstrukturiertes Interview für BDD und SAD, das von derselben geschulten klinischen Psychologin durchgeführt wurde, sind einige methodische Einschränkungen zu berücksichtigen. Erstens handelte es sich um eine monozentrische Studie mit einer insgesamt begrenzten Teilnehmerzahl. Zweitens ließ die Heterogenität der aktuellen Behandlungsregime keine eindeutigen Analysen zu, sodass keine belastbaren Aussagen über den Zusammenhang zwischen Therapie und dem Vorliegen von schambezogenen Störungen getroffen werden können. Drittens könnte die Rekrutierung von Teilnehmern aus einer universitären Ambulanz die externe Validität einschränken, sodass unklar bleibt, ob die berichteten Befunde auf andere Patientengruppen übertragbar sind. Viertens war die gesundheitsbezogene Lebensqualität bei Patienten mit SRD zwar signifikant niedriger, jedoch lagen die absoluten DLQI‐Werte nur bei AD‐Patienten mit SRD über dem Cut‐off‐Wert von 10, der eine starke Beeinträchtigung kennzeichnet. Schließlich stellt das Fehlen einer alters‐ und geschlechtsangepassten Kontrollgruppe aus hautgesunden Personen eine weitere Limitation dar.

Trotz dieser Einschränkungen legen unsere Studie sowie frühere Untersuchungen^26^, ^28^, ^43^ nahe, dass die schambezogenen Störungen BDD und SAD bei dermatologischen Patienten mit AD und Pso häufig vorkommen. Vorausgesetzt, dass unsere Ergebnisse bestätigt werden und dass schambezogene Störungen die Lebensqualität beeinträchtigen, wird zukünftige Forschung zeigen, ob die frühzeitige Erkennung und die angemessene Behandlung von SRD geeignet sind den Leidensdruck der Patienten zu lindern und ihre Lebensqualität zu verbessern.

## DANKSAGUNG

Wir danken allen Patienten, Ärztinnen und Ärzten, die an der Studie teilgenommen haben.

Open access Veröffentlichung ermöglicht und organisiert durch Projekt DEAL.

## FINANZIERUNG

C.W. wurde durch eine Forschungsunterstützung der Almirall Hermal GmbH finanziert. S.E. wird von der Deutschen Forschungsgemeinschaft (DFG: EM 68/13‐1; EM 68/15‐1; GRK 2901/1), dem Bundesministerium für Bildung und Forschung (BMBF: 16GW0345), der Europäischen Union (HORIZON‐MSCA‐2022‐DN‐01, Proposal Number 101118430; PlasTHER COST Action CA20114), dem Bundesministerium für Wirtschaft und Klimaschutz (BMWK: 03TN0019B), dem Ministerium für Wirtschaft, Infrastruktur, Tourismus und Arbeit Mecklenburg‐Vorpommern (TBI‐V‐1–349‐VBW‐120) sowie durch den Europäischen Fonds für regionale Entwicklung (EFRE: GSH‐20–0054) unterstützt. Die Förderinstitutionen hatten keinen Einfluss auf das Studiendesign, die Datenerhebung, ‐analyse oder ‐interpretation, das Verfassen des Manuskripts oder die Entscheidung zur Einreichung des Artikels zur Veröffentlichung.

## INTERESSENKONFLIKT

C.W. hat Beraterhonorare von Almirall erhalten. S.E. hat Honorare oder Reisekostenzuschüsse von Abbvie, Almirall, Amgen, Bristol‐Meyers Squibb, Cinogy, Galderma, Janssen, LEO, Malinckrodt, Mayne Genzyme Corporation, Merck Sharp & Dohme, Novartis, Oncobeta, Pierre‐Fabre, Pfizer, RheaCell, Sanofi, SolGel, SUN Pharma, Teion und UCB erhalten. A.T. hat Honorare und/oder Reisekostenzuschüsse von AbbVie, Almirall, Boehringer Ingelheim, Bristol‐Meyers Squibb, Galderma, GlaxoSmithKline, Janssen, Kyowa Kirin, LEO, Lilly, Merck Sharp & Dohme, Novartis, Pfizer, Pierre Fabre, Recordati Rare Diseases, Sanofi und UCB sowie Forschungsförderungen von Almirall und Cinogy erhalten. Die übrigen Autoren erklären keine Interessenkonflikte.

## Supporting information



Supplementary information
