# Supplementary material for: Schambezogene Störungen bei Patienten mit atopischer Dermatitis und Psoriasis – eine explorative, Querschnitts‐Interviewstudie zu Prävalenz und Korrelaten körperdysmorpher Störungen und sozialer Angststörungen
Source: J Dtsch Dermatol Ges. 2026 Feb 5;24(2):196–206. [Article in German] doi: 10.1111/ddg.15892_g (PMC12875169; doi:10.1111/ddg.15892_g)
Supplement: Supplementary file 1 — Supplementary information [file DDG-24-196-s001.docx]

[[Online-Supplement]]

TABELLE S1 Vergleich von Alter, Geschlecht, Schweregrad und Dauer der Erkrankung sowie Lebensqualität bei Patienten mit chronisch-entzündlichen Hauterkrankungen mit und ohne schambezogene Störungen (SRD; Lebenszeit), stratifiziert nach Schweregrad der Erkrankung (leicht links; mittelschwer bis schwer rechts).

|  | **Schweregrad: leicht (n = 136)** | | | |  | **Schweregrad: mittelschwer bis schwer (n = 15)** | |  |
| --- | --- | --- | --- | --- | --- | --- | --- | --- |
| ***Gesamtstichprobe*** | ***SRD +*** | ***SRD –*** | ***Statistik*** | |  | ***SRD +*** | ***SRD –*** |  |
| n ( %) | 43 (31,6) | 93 (68,4) | ***U/ χ^2^*** | ***p*** |  | 5 (33,3) | 10 (60,7) |  |
| Alter (in Jahren; MW ± SD) | 38,2 ± 16,3 | 48,8 ± 14,9 | 1239,5 | *< 0,001* |  | 36,6 ± 13,2 | 48,8 ± 19,3 |  |
| Frauen | 27 (62,8 %) | 30 (32,3 %) | 11,259 | *< 0,001* |  | 3 (60,0 %) | 5 (50,0 %) |  |
| sichtbare Läsion | 26 (60,5 %) | 54 (59,3 %) | 0,015 | 0,901 |  | 5 (100,0 %) | 9 (90,0 %) |  |
| Krankheitsdauer (in Jahren; MW ± SD) | 20,7 ± 15,4 | 24,8 ± 17,0 | 1679,5 | 0,187 |  | 13,0 ± 12,2 | 21,4 ± 19,8 |  |
| DLQI | 5,81 ± 6,28 | 3,83 ± 5,59 | 1461,5 | *0,011* |  | 15,50 ± 8,27 | 9,00 ± 5,31 |  |
| ***AD-Teilstichprobe*** |  |  |  |  |  |  |  |  |
| n ( %) | 14 (28,6) | 35 (71,4) |  |  |  |  |  |  |
| Alter (in Jahren; MW ± SD) | 31,3 ± 11,7 | 43,2 ± 17,1 | 131,5 | *0,012* |  | 28,0 ± 12,7 | 51,0 ± 16,7 |  |
| Frauen | 10 (71,4 %) | 13 (37,1 %) | 4,720 | *0,030* |  | 1 (50,0 %) | 2 (50,0 %) |  |
| EASI (MW ± SD) | 2,54 ± 2,60 | 4,07 ± 4,03 | 197,0 | 0,287 |  | 27,35 ± 15,34 | 20,75 ± 7,30 |  |
| sichtbare Läsion | 5 (35,7 %) | 7 (21,2 %) | 1,087 | 0,297 |  | 2 (100,0 %) | 3 (75,0 %) |  |
| Krankheitsdauer (in Jahren; MW ± SD) | 28,1 ± 13,5 | 29,1 ± 16,6 | 217,0 | 0,745 |  | 8,5 ± 12,0 | 19,0 ± 22,0 |  |
| DLQI | 7,36 ± 8,24 | 4,94 ± 5,05 | 216,0 | 0,518 |  | 22,50 ± 2,12 | 9,25 ± 7,46 |  |
| ***PSO-Teilstichprobe*** |  |  |  |  |  |  |  |  |
| n ( %) | 29 (33,3) | 58 (66,7) |  |  |  |  |  |  |
| Alter (in Jahren, MW ± SD) | 41,5 ± 17,3 | 51,7 ± 12,5 | 543,5 | *0,007* |  | 42,3 ± 11,9 | 47,3 ± 22,2 |  |
| Frauen | 17 (58,6 %) | 17 (29,3 %) | 6,976 | *0,008* |  | 2 (66,7 %) | 3 (50,0 %) |  |
| PASI (MW ± SD) | 2,05 ± 2,68 | 1,71 ± 2,57 | 720,5 | 0,266 |  | 22,50 ± 8,58 | 15,55 ± 10,21 |  |
| sichtbare Läsion | 17 (58,6 %) | 28 (48,3 %) | 0,829 | 0,363 |  | 3 (100,0 %) | 6 (100,0 %) |  |
| Krankheitsdauer (in Jahren;  MW ± SD) | 17,1 ± 15,2 | 22,3 ± 16,8 | 691,0 | 0,176 |  | 17,5 ± 14,8 | 23,0 ± 20,2 |  |
| DLQI | 5,07 ± 5,09 | 3,16 ± 5,83 | 526,5 | *0,004* |  | 8,50 ± 2,12 | 8,83 ± 4,17 |  |

*Abk.:* DLQI, dermatologischer Lebensqualitätsindex; EASI, *eczema area severity index*; MW, Mittelwert; PASI, *psoriasis area severity index*; SD, Standardabweichung

TABELLE S2 Vergleich von Alter, Geschlecht, Schweregrad und Dauer der Erkrankung sowie Lebensqualität bei Patienten mit chronisch-entzündlichen Hauterkrankungen mit und ohne körperdysmorphe Störung (BDD; Lebenszeit), stratifiziert nach Schweregrad der Erkrankung (leicht links; mittelschwer bis schwer rechts).

|  | **Schweregrad: leicht (n = 136)** | | | |  | **Schweregrad: mittelschwer bis schwer (n = 15)** | |  |
| --- | --- | --- | --- | --- | --- | --- | --- | --- |
| ***Gesamtstichprobe*** | ***BDD +*** | ***BDD –*** | ***Statistik*** |  |  | ***BDD +*** | ***BDD –*** |  |
| n ( %) | 37 (27,2) | 99 (72,8) | ***U/ χ^2^*** | ***p*** |  | 3 (20,0) | 12 (80,0) |  |
| Alter (in Jahren; MW ± SD) | 38,0 ± 16,8 | 47,9 ± 14,9 | 1162,5 | *< 0,001* |  | 30,0 ± 16,5 | 48,4 ± 17,9 |  |
| Frauen | 25 (67,6 %) | 32 (32,3 %) | 13,742 | *< 0,001* |  | 2 (66,7 %) | 6 (50,0 %) |  |
| sichtbare Läsion | 21 (56,8 %) | 59 (60,8 %) | 0,184 | 0,668 |  | 3 (100,0 %) | 11 (91,7 %) |  |
| Krankheitsdauer (in Jahren; MW ± SD) | 22,4 ± 15,4 | 23,9 ± 17,0 | 1724,5 | 0,727 |  | 22,5 ± 7,8 | 18,4 ± 19,3 |  |
| DLQI | 5,62 ± 6,55 | 4,02 ± 5,57 | 1473,5 | 0,076 |  | 15,50 ± 12,02 | 10,08 ± 5,92 |  |
| ***AD-Teilstichprobe*** |  |  |  |  |  | ***n = 6*** |  |  |
| n ( %) | 13 (26,5) | 36 (73,5) |  |  |  | 1 (16,7) | 5 (83,3) |  |
| Alter (in Jahren, MW ± SD) | 31,7 ± 12,0 | 42,7 ± 17,1 | 136,0 | *0,026* |  | 19,0 | 48,2 ± 15,8 |  |
| Frauen | 10 (76,9 %) | 13 (36,1 %) | 6,387 | *0,011* |  | 1 (100,0 %) | 2 (40,0 %) |  |
| EASI (MW ± SD) | 2,59 ± 2,70 | 4,00 ± 4,00 | 189,0 | 0,307 |  | 38,20 | 19,90 ± 6,60 |  |
| sichtbare Läsion | 8 (61,5 %) | 27 (79,4 %) | 1,580 | 0,209 |  | 1 (100,0 %) | 4 (80,0 %) |  |
| Krankheitsdauer (in Jahren;  MW ± SD | 28,5 ± 14,0 | 28,9 ± 16,4 | 213,0 | 0,849 |  | 17,0 | 15,2 ± 20,9 |  |
| DLQI | 6,92 ± 8,41 | 5,17 ± 5,15 | 222,0 | 0,785 |  | 24,00 | 11,60 ± 8,32 |  |
| ***PSO- Teilstichprobe*** |  |  |  |  |  | ***n = 9*** |  |  |
| n ( %) | 24 (27,6) | 63 (72,4) |  |  |  | 2 (22,2 %) | 7 (77,8 %) |  |
| Alter (in Jahren, MW ± SD) | 41,3 ± 18,3 | 50,9 ± 12,7 | 515,5 | *0,022* |  | 35,5 ± 2,1 | 48,6 ± 13,5 |  |
| Frauen | 15 (62,5 %) | 19 (30,2 %) | 7,635 | *0,006* |  | 1 (50,0 %) | 4 (57,1 %) |  |
| PASI (MW ± SD) | 2,02 ± 2,56 | 1,75 ± 2,63 | 661,0 | 0,355 |  | 27,15 ± 4,17 | 15,21 ± 9,36 |  |
| sichtbare Läsion | 13 (54,2 %) | 32 (50,8 %) | 0,079 | 0,778 |  | 2 (100,0 %) | 7 (100,0 %) |  |
| Krankheitsdauer (in Jahren;  MW ± SD) | 19,1 ± 15,4 | 21,1 ± 16,8 | 722,0 | 0,747 |  | 28,0 ± 5,2 | 20,7 ± 19,4 |  |
| DLQI | 4,92 ± 5,36 | 3,37 ± 5,73 | 546,5 | *0,042* |  | 7,00 ± 2,74 | 9,00 ± 3,83 |  |

*Abk.:* DLQI, dermatologischer Lebensqualitätsindex; EASI, *eczema area severity index*; MW, Mittelwert; PASI, *psoriasis area severity index*; SD, Standardabweichung

TABELLE S3 Vergleich von Alter, Geschlecht, Schweregrad und Dauer der Erkrankung sowie Lebensqualität bei Patienten mit chronisch-entzündlichen Hauterkrankungen mit und ohne soziale Angststörung (SAD; Lebenszeit), stratifiziert nach Schweregrad der Erkrankung (leicht links; mittelschwer bis schwer rechts).

|  | **Schweregrad: leicht (n = 136)** | | | |  | **Schweregrad: mittelschwer bis schwer (n = 15)** | |  |
| --- | --- | --- | --- | --- | --- | --- | --- | --- |
| ***Gesamtstichprobe*** | ***SAD +*** | ***SAD –*** | ***Statistik*** |  |  | ***SAD +*** | ***SAD –*** |  |
| n ( %) | 22 (16,2) | 114 (83,8) | ***U/ χ^2^*** | ***p*** |  | 4 (33,3) | 11 (73,3) |  |
| Alter (in Jahren; MW ± SD) | 35,7 ± 14,2 | 47,1 ± 15,8 | 745,0 | *0,003* |  | 36,5 ± 15,2 | 47,7 ± 18,6 |  |
| Frauen | 14 (63,6 %) | 43 (37,7 %) | 5,088 | *0,024* |  | 3 (75,0 %) | 5 (45,5 %) |  |
| sichtbare Läsion | 13 (59,1 %) | 67 (59,8 %) | 0,004 | 0,949 |  | 4 (100,0 %) | 10 (90,9 %) |  |
| Krankheitsdauer (in Jahren; MW ± SD) | 18,0 ± 14,9 | 24,5 ± 16,7 | 950,0 | 0,090 |  | 13,0 ± 12,2 | 21,4 ± 19,8 |  |
| DLQI | 5,64 ± 6,64 | 4,22 ± 5,71 | 1034,5 | 0,188 |  | 15,50 ± 8,27 | 9,00 ± 5,31 |  |
| ***AD-Teilstichprobe*** |  |  |  |  |  | ***n = 6*** |  |  |
| n ( %) | 7 (14,3) | 42 (85,7) |  |  |  | 2 (33,3 %) | 4 (66,7 %) |  |
| Alter (in Jahren, MW ± SD) | 30,7 ± 12,7 | 41,3 ± 16,7 | 82,5 | 0,065 |  | 28,0 ± 12,7 | 51,0 ± 16,7 |  |
| Frauen | 4 (57,1 %) | 19 (45,2 %) | 0,341 | 0,559 |  | 1 (50,0 %) | 2 (50,0 %) |  |
| EASI (MW ± SD) | 3,46 ± 3,32 | 3,66 ± 3,82 | 143,0 | 0,909 |  | 27,35 ± 15,34 | 20,75 ± 7,30 |  |
| sichtbare Läsion | 6 (85,7 %) | 29 (72,5 %) | 0,547 | 0,459 |  | 2 (100,0 %) | 3 (75,0 %) |  |
| Krankheitsdauer (in Jahren;  MW ± SD | 28,0 ± 14,5 | 29,0 ± 16,0 | 130,0 | 0,765 |  | 8,5 ± 12,0 | 19,0 ± 22,0 |  |
| DLQI | 10,00 ± 9,73 | 4,90 ± 5,14 | 102,0 | 0,208 |  | 22,50 ± 2,12 | 9,25 ± 7,46 |  |
| ***PSO-Teilstichprobe*** |  |  |  |  |  | ***n = 9*** |  |  |
| n ( %) | 15 (17,2) | 72 (82,8) |  |  |  | 2 (22,2 %) | 7 (77,8 %) |  |
| Alter (in Jahren, MW ± SD) | 38,1 ± 14,6 | 50,4 ± 14,3 | 299,0 | *0,007* |  | 45,0 ± 15,6 | 45,9 ± 20,7 |  |
| Frauen | 10 (66,7 %) | 24 (33,3 %) | 5,794 | *0,016* |  | 2 (100,0 %) | 3 (42,9 %) |  |
| PASI (MW ± SD) | 1,45 ± 2,90 | 1,90 ± 2,54 | 435,0 | 0,226 |  | 18,70 ± 7,78 | 17,63 ± 10,82 |  |
| sichtbare Läsion | 7 (46,7 %) | 38 (52,8 %) | 0,186 | 0,667 |  | 2 (100,0 %) | 7 (100,0 %) |  |
| Krankheitsdauer (in Jahren;  MW ± SD) | 13,3 ± 13,1 | 22,1 ± 16,7 | 374,0 | 0,062 |  | 17,5 ± 14,8 | 23,0 ± 20,2 |  |
| DLQI | 3,60 ± 3,42 | 3,83 ± 6,02 | 447,5 | 0,287 |  | 8,50 ± 2,12 | 8,83 ± 4,17 |  |

*Abk.:* DLQI, dermatologischer Lebensqualitätsindex; EASI, *eczema area severity index*; MW, Mittelwert; PASI, *psoriasis area severity index*; SD, Standardabweichung

TABELLE S4 Vergleich der Lebenszeitprävalenz von schambezogenen Störungen (SRD), körperdysmorpher Störung (BDD) und sozialer Angststörung (SAD) bei Patienten, die mit Biologika behandelt wurden im Vergleich zu Patienten ohne Biologika (links), sowie bei Patienten, die mit Biologika behandelt wurden im Vergleich zu Patienten, die ausschließlich topische Medikamente erhielten (rechts).

| **Gesamtstichprobe** | **Biologika +** | **Biologika –** | **Statistik** |  |  | **Biologika +** | **Nur topisch** | **Statistik** |  |
| --- | --- | --- | --- | --- | --- | --- | --- | --- | --- |
| **n** ( %) | 91 (60,3) | 60 (39,7) | ***U/ χ^2^*** | ***p*** |  | 91 (79,1) | 24 (20,9) | ***U/ χ^2^*** | ***p*** |
| SRD | 33 (36,3) | 15 (25,0) | 2,116 | 0,146 |  | 33 (36,3) | 5 (20,8) | 2,044 | 0,153 |
| BDD | 29 (31,9) | 11 (18,3) | 3,402 | 0,065 |  | 29 (31,9) | 3 (12,5) | 3,547 | 0,060 |
| SAD | 17 (18,7) | 9 (15,0) | 0,344 | 0,558 |  | 17 (18,7) | 3 (12,5) | 0,505 | 0,477 |
| ***AD-Teilstichprobe*** | 35 (63,6) | 20 (36,4) |  |  |  | 35 (77,8) | 10 (22,2) |  |  |
| SRD | 11 (31,4) | 5 (25,0) | 0,255 | 0,614 |  | 11 (31,4) | 2 (20,0) | 0,495 | 0,482 |
| BDD | 10 (28,6) | 4 (20,0) | 0,493 | 0,483 |  | 10 (28,6) | 2 (20,0) | 0,292 | 0,589 |
| SAD | 5 (14,3) | 4 (20,0) | 0,304 | 0,582 |  | 5 (14,3) | 1 (10,0) | 0,124 | 0,725 |
| ***PSO-Teilstichprobe*** | 56 (58,3) | 40 (41,7) |  |  |  | 56 (80,0) | 14 (20,0) |  |  |
| SRD | 22 (39,3) | 10 (25,0) | 2,143 | 0,143 |  | 22 (39,3) | 3 (21,4) | 1,556 | 0,212 |
| BDD | 19 (33,9) | 7 (17,5) | 3,189 | 0,074 |  | 19 (33,9) | 1 (7,1) | 3,938 | 0,054 |
| SAD | 12 (21,4) | 5 (12,5) | 1,276 | 0,259 |  | 12 (21,4) | 2 (14,3) | 0,357 | 0,550 |

*Abk.:* SRD, schambezogene Störung; BDD, körperdysmorphe Störung; SAD, soziale Angststörung
